# Training needs and curriculum of continuing medical education among general practitioners in Tibet, China: A cross-sectional survey

**DOI:** 10.3389/fpubh.2022.914847

**Published:** 2022-10-11

**Authors:** Kang An, Lin Zhang, Runjuan Qiao, Caizheng Li, Qian Zhong, Yiru Ma, Xin Rao, Tingrui Mao, Feng Liu, Qiang Zhuo, Yi She, Shuangqing Li, Qiaoli Su

**Affiliations:** ^1^General Practice Ward/International Medical Center Ward, General Practice Medical Center, National Clinical Research Center for Geriatrics, West China Hospital, Sichuan University, Chengdu, China; ^2^Department of Endocrinology and Metabolism, West China Hospital, Sichuan University, Chengdu, China; ^3^Department of General Practice, People's Hospital of Lhasa, Lhasa, China; ^4^Fangcao Community Health Service Center, Chengdu, China; ^5^Jincheng Community Health Service Center, Chengdu, China

**Keywords:** continuing medical education, primary care, curriculum development, training needs, general practice

## Abstract

**Background:**

Uneven distribution of health resources is higher in Tibet than in other regions. The development of core professional capability for general practitioners (GPs) is the main goal of continuing medical education (CME) training programs.

**Aim:**

This study aimed to explore the needs of CME for GPs and provide advice for the development of policy, practice, and CME curriculums.

**Methods:**

We conducted a cross-sectional online survey among GPs in Tibet Autonomous Region, China. We designed an online questionnaire including the demographic section, training contents, and training formats about CME.

**Results:**

A total of 108 questionnaires were included in this study. Notably, 79 (73.15%) were women and 56 participants (51.85%) were working in primary care settings. We developed a curriculum priority: first-choice, major alternatives, and secondary considerations. The topics identified as first-choice for CME were related to “cardiovascular disease” (85.19%), “respiratory disease” (81.48%), and “digestive disease” (80.56%). Major alternatives included two essential knowledge and eight clinical skill items. We rated 10 items as secondary considerations. Only 39.81% ranked mental health as an essential priority; bedside teaching (51.85%) was the first choice.

**Conclusion:**

We presented priority areas identified in this study to focus on CME for GPs in Tibet. The 23 topics may reflect the features of general practice, which increasingly require common disease management skills, while a demand-oriented curriculum and staged training plans should be adopted. CME programs should be adapted dynamically to respond to evolving needs.

## Introduction

Healthy China 2030 is a breakthrough in the effort to strengthen national health policy and focus on the delivery of comprehensive life-cycle healthcare for the Chinese people (1). This national strategy aimed to provide access to five competent general practitioners (GPs) for every 10,000 residents by 2030 (2). Pairing assistance between the eastern and western regions is a major decision in China to promote resource complementarity and exchange of talents (3). Notably, China has experienced considerable difficulties with the training and cultivation of GPs in resource-poor areas. Over the past 10–15 years, reform of medicine and the healthcare system is providing enough resources to rapidly increase the number of future GPs in underdeveloped rural areas. In 2018, the Tibet Autonomous Region (Tibet) announced incentive policies to recruit additional GPs to work in primary care (4).

In 2020, the Health Commission of Tibet autonomous region put forward a plan: pairing-up assistance from tertiary hospitals across the country, covering all 74 counties and districts in Tibet. Under the policy of pairing-up assistance for Tibet, some excellent hospitals in other provinces were designated to assist in specific areas of Tibet. Such assistance programs from other provincial-level regions enabled the exchange of traditional Chinese medicine, Tibetan medicine, and western medicine and improved accessibility and usability of primary care. Pairing-up assistance belonged to the category of continuing medical education (CME) and was generally assessed by a self-assessment questionnaire. Compared with the “5 + 3” mode and other rigorous programs for training GPs in the US and European countries (5, 6), the CME training in remote Tibetan villages may be a sped-up solution. At present, Tibet has implemented a series of CME training programs, such as transition, short courses, and on-the-job training at hospitals that offer pairing-up assistance (4, 7, 8). In general, training contents include two modules, namely, essential knowledge and clinical skills (9). Consequently, a large number of Tibet GPs are taking advantage of educational opportunities.

Continuing medical education accounted for a large portion of the process of lifelong learning that GPs could use to update knowledge and develop skills. A comprehensive CME program is essential for GPs to maintain high standards of practice (10). CME is an integral part of a lifelong learning process in which GPs constantly acquire new knowledge, skills, and attitudes. A highly effective channel in developing training programs is the assessment of trainee needs. The purpose of educational needs assessments is to identify the gap between what is known and what should be known (11, 12). Individual, work unit, and organization factors should receive high priority in framing government policy (11).

Existing evidence suggests that programs based on well-designed needs assessments are more likely to cause changes in trainee behavior (13, 14). Training formats of CME comprise medical conferences, professional meetings, intensive courses, oral presentations, bedside teaching, and more recently, online courses (15). Diverse learning methods tend to suit different doctors and identified learning needs (14). Different approaches may be necessary at various stages of the learning process. However, few studies have formally explored evidence-based recommendations to improve training satisfaction and participation enthusiasm (16, 17).

To resolve a shortage of GPs and the complex medical needs of an aging population, the design of higher-quality general practice courses and standards has received increasing attention recently (18, 19). The curriculum and practice standards for one provincial-level administrative region do not necessarily wholly relate to the needs of another. The arrangement of medical ability training for GPs in China lacks evidence-based support and is influenced by the subjective opinions of managers. We conducted a questionnaire survey, which covered nine different community health centers within a medical consortium, to collect data about training needs for GPs in the Chinese community health center and formulate a demand-oriented training to provide a reference for policymakers in education (9).

General practitioners in Tibet are few in number and mostly work in rural areas. They rarely travel to hospitals in major cities to accept training due to transportation costs, work pressure, and low-income concerns (20). A demand-oriented training plan and preferred training formats can help bridge the access gap by mitigating the cost and time concerns of Tibet GPs. In this survey, we extended our search through optimized questionnaires. Moreover, this study aimed to determine the demand-oriented curriculum and learning method preferences of GPs in Tibet, given that previous studies suggest that varying GP types have different CME training needs (20).

## Methods

### Study design

The Department of General Practice of West China Hospital of Sichuan University was launched in January 2020 and funded for 3 years. It provided pairing-up support for Tibet to improve medical services. The project covered most hospitals and community health services across Lhasa, as well as parts of six prefecture-level cities in the region.

We conducted a cross-sectional online study, using convenience and snowball sampling approaches to recruit GPs from partner organizations and the network of general practice in Western China through a questionnaire web link. Furthermore, we sent two reminder text messages. Tibet GPs were characterized by large dispersion, low density, and small scale, which led to difficulty in recruiting. Snowballing sampling is considered an effective and efficient approach to build a sample through the Internet (21). We invited participants to share the web link with their colleagues on social media (e.g., WeChat, QQ, and other internet platforms). As participants re-shared the link, it led to snowball sampling. Eligible participants included GPs who were registered in a general practice specialty at all levels of the health system in Tibet, recognize Chinese characters, and were willing to participate in this study.

We collected data from 1 January 2021 to 28 February 2022 and invited participants to complete the questionnaire within 1 week, personally, or *via* e-mail.

### Quality control

We took quality control measures to ensure data quality. Participants could only submit their responses once and could not edit their responses after submission. All survey items had to be completed on submission. We eliminated survey data with logical errors.

### Description of questionnaire

We based the training needs assessment questionnaire about CME on an established questionnaire developed by our previous findings (9). The tool allowed us to assess training needs and format to guide the development of curriculums, determine gaps in needs and training programs, and help establish training and education priorities. The questionnaire was justified by an advisory panel, including educationalists, policymakers, GPs sent to support Tibet, and methodologists. Throughout the modification process, the multidisciplinary team performed a pilot survey to check the length of the questionnaire, which was not more than 15 min, along with its fluency, feasibility, and understandability.

We divided the questionnaire into three sections, namely, demographic (6 questions), training contents (23 questions), and training formats (1 question) (refer to the Supplementary material “Training needs assessment questionnaire in continuing medical education”). First, we collected basic demographic information, including age, gender, years of practice, status of a university degree, workplace (hospital, primary care), and whether they were engaged in chronic disease management (yes/no). Section Methods of the survey inquired about 23 skills in two core professional capability subscale domains, namely, essential knowledge (11 items) and clinical skills (12 items). For the 23 skills, respondents indicated the level of training needs using a five-point Likert scale 1–not a priority; 2–low priority; 3–medium priority; 4–high priority; and 5–essential priority). They were ascribed as not in of need training for topics where they selected “Not a priority” or “Low priority.” Scores 1 or 2 in the training need index indicates no need for training in the specific area. Scores of 3 and greater indicate the need for training in the specific area. High total scores indicated higher degrees of training need. We added the score for each item to form the subscale score, which ranges between 11 and 55 and 12 and 60. In section Results, we asked GPs to choose a training format they preferred from a list, including intensive courses, oral presentations, bedside teaching, and online instruction, to determine the best training format through education and learning.

### Ethics

The ethical committee of West China Hospital, Sichuan University, Chengdu, China (No. 2021-1735) approved this study. We informed all participants about the purpose of this study. We also informed them that the data would remain anonymous and confidential. We considered data submission *via* a questionnaire as implied consent.

### Data analysis

We used descriptive statistics to explore qualitative data. We presented total scores as median (interquartile range, IQR) as they were not distributed normally. We calculated differences in total scores with the Kruskal–Wallis H and Mann–Whitney U tests between each group; *P* < 0.05 (two-tailed) indicated statistical significance. Moreover, we applied a Bonferroni correction for multiple comparisons type I errors. Microsoft Excel was used to create the graphical representations. We analyzed all data using SPSS version 24.0 (IBM Corporation).

## Results

### Demographic characteristics

Of the 110 questionnaires that were returned, a total of 108 questionnaires were included in the analyses since 2 were logical errors. Table 1 summarized a complete description of the participants. More than half of the samples were women (73.15%), with a university degree (75.00%). Notably, 51.85% (*n* = 56) of responders worked at primary care institutes, 40.74% (*n* = 44) were <30 years, and 71.30% (*n* = 77) practiced for more than 5 years. The minority (*n* = 33, 30.56%) engaged in chronic disease management.

**Table 1:** Participants' characteristics and training need variables.

	**Total**		**Essential knowledge**		**Clinical skills**	
**Variable**	***n***	**%**	**Median (25–75%)**	***p*-Value**	**Median (25–75%)**	***p*-Value**
Age group (years)				0.17*		0.87*
−29	44	40.74	50.00 (42.25, 52.75)		56.00 (52.00, 59.00)	
30–39	56	51.85	50.00 (46.00, 54.00)		55.00 (50.25, 59.00)	
40+	8	7.41	53.00 (50.25, 53.75)		57.50 (43.75, 60.00)	
Gender				0.72**		0.32**
Male	29	26.85	50.00 (45.00, 54.00)		56.00 (46.50, 59.00)	
Female	79	73.15	50.00 (46.00, 53.00)		56.00 (52.00, 59.00)	
Year of practice (years)				0.04*		0.13*
<5	31	28.70	50.00 (40.00, 52.00)		55.00 (50.00, 58.00)	
5–9	39	36.11	52.00 (47.00, 55.00)		58.00 (51.00, 60.00)	
>9	38	35.19	50.00 (46.00, 53.00)		54.50 (48.75, 59.00)	
Education level				0.06**		0.35**
No university degree	27	25.00	52.00 (46.00, 55.00)		56.00 (52.00, 60.00)	
University degree	81	75.00	50.00 (46.00, 53.00)		55.00 (50.50, 59.00)	
Workplace				0.43**		0.92**
Hospital	52	48.15	50.00 (45.25, 53.00)		56.00 (50.25, 59.00)	
Primary care	56	51.85	51.00 (46.25, 54.00)		55.50 (51.25, 59.00)	
Chronic disease management				0.30**		0.78**
Yes	33	30.56	51.00 (44.50, 55.00)		56.00 (51.00, 59.50)	
No	75	69.44	50.00 (46.00, 53.00)		56.00 (51.00, 59.00)	

* Kruskal-Wallis test;

** Mann-Whitney test.

### Training needs assessment

The Cronbach's alpha coefficients for individual scales were 0.87 and 0.92. The total training need was the sum of all subscale scores between 23 and 115. The overall Cronbach's alpha coefficient was 0.94, which demonstrated appropriate reliability. The median scores for the total, essential knowledge, and clinical skills were 105 (96.25–112.00), 50 (46.00–53.75), and 56 (51–59), respectively. All items received a median scale rating of 4 or above on the five-point scale. The Kruskal-Wallis test showed a statistically significant difference between the year of practice on essential knowledge scores (*H* = 6.31, *P* = 0.04). After the Bonferroni correction, participants with 5–9 years of practice scored more than those with <5 years of practice [50.00 (40.00, 52.00) vs. 52.00 (47.00, 55.00), adjusted *P* = 0.04]. None of the two subscale scores differed between other subgroups (refer to Table 1).

### Evaluation of training priorities

**Figure 1:**
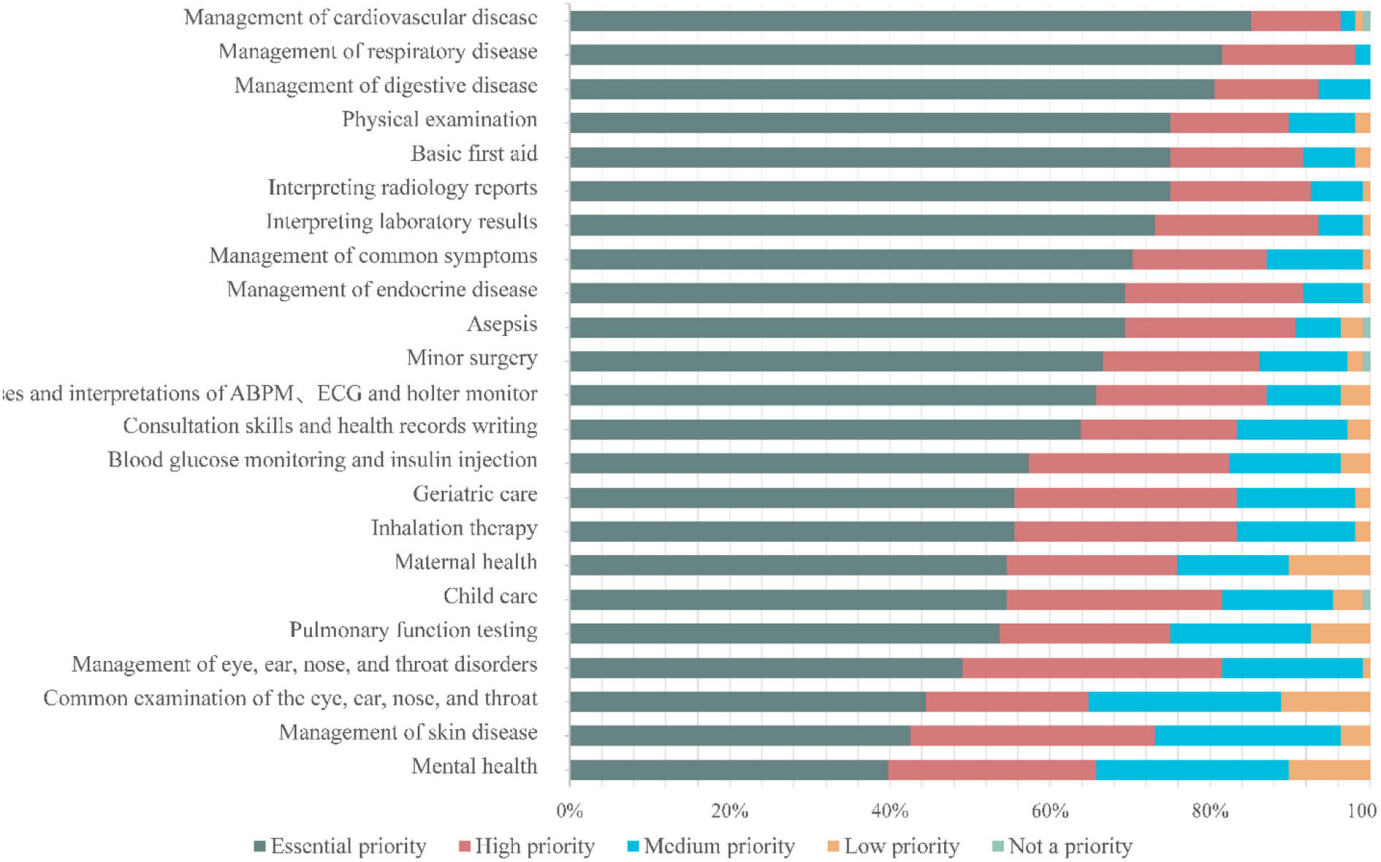
Participants' priorities of training needs.

**Figure 2:**
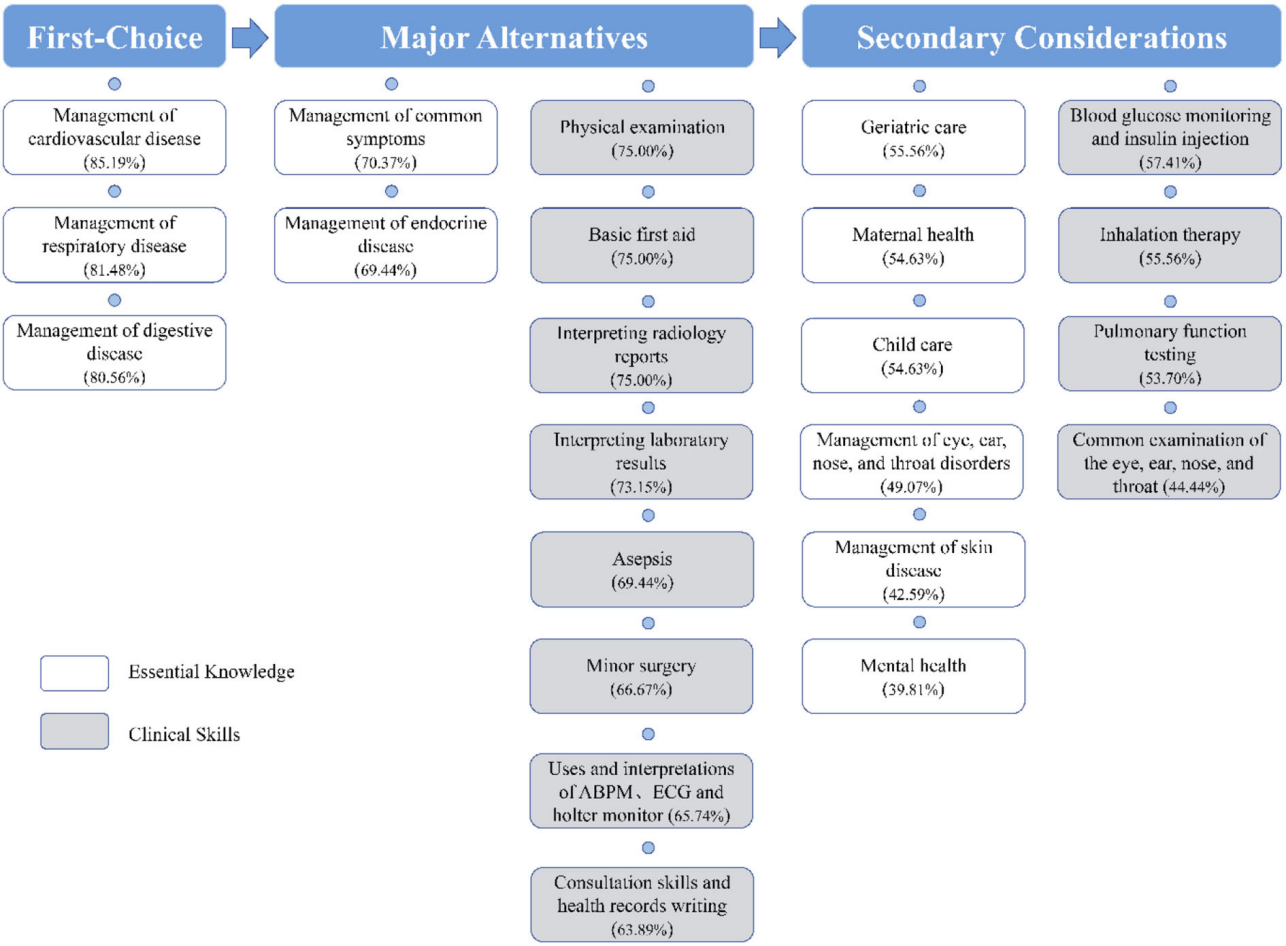
Flowchart depicting the priority level.

Based on the proportion of participants reporting scores of 5, we ranked the priority from the most to the least needed training area. An appropriate cutoff would ensure that the proper number of skills (17) were chosen. However, we selected 20% points to develop a curriculum priority–first-choice and major alternatives, secondary considerations–to ensure that the number would not be unexplainable. We also used this cutoff in a previous study (14). In this section, we first described the self-assessment of the priorities of the 23 items included in the questionnaire. Figure 1 provides information about the relative priorities of each item. According to the priority of the items, we set up three level modules to construct a systematic multi-stage training course. Across the study population, management related to “cardiovascular disease” (85.19%), “respiratory disease” (81.48%), and “digestive disease” (80.56%) was identified as the first choice. Ten items were rated as major alternatives. Regarding essential knowledge, participants considered the following: management related to “common symptoms” and “endocrine disease.” As for the clinical skills in descending order, the topics were “physical examination,” “basic first aid,” “interpreting radiology reports,” “interpreting laboratory results,” “asepsis,” “minor surgery,” “uses and interpretations of ABPM, ECG, and Holter monitor,” and “consultation skills and health records writing.” Secondary considerations included six essential knowledge and four clinical skill items. Mental health was the least demand item with only 39.81% (43/108) of participants considering it essential. Figure 2 depicts a flowchart of priority levels with percentages of the essential priority.

### Training format

The choices of participants regarding training formats were diverse. The vast majority of participants choose face-to-face instruction as the first choice, including bedside teaching (51.85%), intensive courses such as classroom-based (35.19%), and oral presentations such as conferences and lectures (3.70%). Moreover, online instructions were the first choice of 9.26% of participants.

## Discussion

This is the first study to address the training needs of Tibet GPs in the field of clinical practice. A wide geographical representation is a strength of our survey. Participants came from one area and five cities. In this study, we developed a curriculum priority: first-choice, major alternatives, and secondary considerations. Our findings underscore the topics and training formats that GPs believe are the top priority for future CME projects. The training priorities can serve as a practical reference for making CME curriculums. Moreover, this research can assist policymakers in providing demand-oriented curriculum and staged training plans for GPs.

In this research, GPs expected almost all the training contents. The demand for essential knowledge training of participants with <5 years of practice was significantly higher than those practicing for 5–9 years, which may be related to clinical experience. There is no significant difference in the training needs between other groups, which may be attributed to the fact that GPs may have a vital requirement for continuing education and a desire for better career development. Furthermore, almost (>80%) identified training in cardiovascular, respiratory, and digestive disease management as their top priorities (22). Studies are consistent with previous studies which show that many physicians consider further education essential to improve chronic disease management and highly value these programs (23). Some common reasons result in the similarity of need: due to fewer types of disease, GPs were unable to enrich their clinical experience by contacting patients frequently; the majority of GPs lack postgraduate medical education, and their clinical skills are inadequate; the syllabus of CME training required GPs to learn much knowledge in a short time, resulting in diminished the training effect (9, 24–26). The following top priorities in clinical skills are mainly involved: physical examination, basic first aid, and interpreting reports (X, CT, and ultrasound). Although community health services do not have some advanced medical examination instruments, patients commonly consult GPs on radiology reports and laboratory results. Similarly, GPs should be equipped in delivering prehospital care emergency medical services in the community (27).

Only 39.81% ranked mental health as an essential priority. The comparably low priority rate might be associated with the tendency of GPs to ignore the importance of mental health management. The epidemic data from Tibetans of the Qinghai-Tibet Plateau indicate that the prevalence of depression is higher than the overall Chinese population (28). The international agencies and national mental health guidelines consider integrating mental health and primary care as a priority (29–31). In recent years, there have been increasing calls to provide additional support to guide and promote the nationwide implementation of mental health rehabilitation (32). There is strong evidence to demonstrate that primary care is considerably advantaged in providing evidence-based treatment for patients with mental health (33). Some studies indicate that GPs lacked the expertise to effectively engage with patients with mental health problems (34).

Face-to-face instruction seems to be the most popular option among survey participants. Three possible reasons are as follows: participants may have no previous exposure to distance teaching, the “pairing assistance” measures are based on on-site training, and there is a desire for hands-on skills training. We should not underestimate the value of online instruction during the COVID-19 pandemic as many face-to-face courses were canceled. Worrying about deduction from wages, heavy workload, and transportation costs makes it difficult for GPs to make suitable arrangements between work and training (20). Online instruction is considered the potential for being a convenient, feasible, low-cost alternative to face-to-face instruction (35, 36). A qualitative study of the Chinese community revealed that online training is a solution to the contradiction between work and training for GPs (22). Previous research compared four modes of training format, namely, face-to-face, video conference, online, and blended (37). Findings indicate that all four training formats significantly increased the confidence and knowledge (37) of the trainees. However, web-based conferences are hard to consult in time. The curriculum is not connected with reality, lack of interaction, and other shortcomings with a single form and mode.

## Policy implications

The staged training plans according to an order of priority will be necessary. The policy documents stated that the CME training process would be divided into different stages based on the actual time schedule and the local practical conditions (38). The challenges included the time conflict between work and training, deficiencies of qualified GP teaching teams, and a shortage of educational funds (11, 22, 39). In extrapolating our study results to staged training plans, several points should be considered, including the feedback of trainees, faculty resources, training time, and funding for training (40). As training progresses, three “first-choice” items should be given top priority, followed by the 10 “major alternatives” items and 10 “secondary considerations” items. It is recommended that dynamically adjusting the training plans achieves efficient use of education resources, arouse the enthusiasm of both trainers and trainees, and ultimately integrate education for sustainable development. After the completion of the CME training, it is necessary to pay attention to the effective evaluation and feedback of the training plan.

We call for additional efforts from the National Health Commission and Continuing Medical Education Committee to improve the knowledge and practices of GPs. Chinese health policymakers should formulate evidence-based CME training programs (41, 42). Blended learning is a combination of online with face-to-face training, which allowed GPs in Tibet to choose their training formats and time. The training format provides the staged training plans with feasibility and convenience. Future strategies should also consider building a standardized CME online course library and repeated education by the network to maintain and increase knowledge (43).

These results present the real-world situation of the need for CME training in China, according to the Tibetan GPs' perspective. Nevertheless, some limitations need to be noted. First, snowballing is a well-established technique for recruiting hard-to-reach groups in the public health field. The sampling approach was appropriate for principal purposes as we sought to prioritize participants that presented higher demands in this study. This procedure offers the following advantages over other sampling methods: it is a low-cost and simple process and makes it possible to GPs in remote areas that are difficult to access. Second, the relatively small sample size of GPs may limit the generalizability of our findings. The possible reason lies in the shortage of Tibetan GPs. Finally, this was a cross-sectional, retrospective study, in which participants' needs for CME training might exist reporting bias. Formal methods, such as in-depth interviews, peer assessment, or objective testing, will help us plan education accordingly.

## Conclusion

This study shows the real status of CME training needs for GPs in Tibet drawing. We provided important insights into a demand-oriented curriculum and staged training plans that are a vital composition of the framework for high-quality CME. This study also highlights that CME training formats should consist of hybrid face-to-face and online instruction. The findings of this study help to understand how to adjust CME training programs better, which may contribute to further educational policy-making and Tibetan GPs practicing in order to optimize the teaching of the main theoretical content and practical skill.

## Data availability statement

The raw data supporting the conclusions of this article will be made available by the authors, without undue reservation.

## Ethics statement

The studies involving human participants were reviewed and approved by Ethics Committee on Biomedical Research, West China Hospital of Sichuan University. Written informed consent for participation was not required for this study in accordance with the national legislation and the institutional requirements.

## Author contributions

This study was conceptualized by KA and QS. The database was organized by RQ, CL, XR, and TM. Data analysis was performed by KA, QZ, and LZ. The manuscript with inputs was drafted by KA, QZ, YM, and LZ and reviewed by KA, LZ, SL, FL, YS, and QS. All authors contributed to drafting or revising the manuscript, have agreed on the journal to which the article will be submitted, gave final approval of the version to be published, and agreed to be accountable for all aspects of the work.

## Funding

This study was supported by the National Clinical Research Center for Geriatrics, West China Hospital, Sichuan University (Grant Number Z2021JC005), Reform of Postgraduate Education, Sichuan University (Grant Number GSSCU2021146), and Sichuan Science and Technology Program (Grant Number 2021YFH0168).

## Conflict of interest

The authors declare that the research was conducted in the absence of any commercial or financial relationships that could be construed as a potential conflict of interest.

## Publisher's note

All claims expressed in this article are solely those of the authors and do not necessarily represent those of their affiliated organizations, or those of the publisher, the editors and the reviewers. Any product that may be evaluated in this article, or claim that may be made by its manufacturer, is not guaranteed or endorsed by the publisher.
