# Supplementary material for: Training needs and curriculum of continuing medical education among general practitioners in Tibet, China: A cross-sectional survey
Source: Front Public Health. 2022 Oct 11;10:914847. doi: 10.3389/fpubh.2022.914847 (PMC9592895; doi:10.3389/fpubh.2022.914847)
Supplement: Supplementary file 1 [file Data_Sheet_1.docx]

**TRAINING NEEDS ASSESSMENT QUESTIONNAIRE IN CONTINUING MEDICAL EDUCATION**

1. **Demographic Information**
2. Age group (years):
3. -29
4. 30-39
5. 40+
6. Gender:
7. Male
8. Female
9. Year of practice (years):
10. <5
11. 5-9
12. ＞9
13. What is your education Level?
14. No university degree
15. University degree
16. Where is your current workplace?
17. Hospital
18. Primary care
19. Are you currently engaged in chronic disease management?
20. Yes
21. No
22. **Training contents**

For the following set of skills, we will inquire about priority needs in specific skills.

On a Scale of 5-Essential priority to 1-Not a priority, how do you assess skills in these areas?

**Part 1. Essential knowledge**

| Skill | Not a priority | Low priority | Medium priority | High priority | Essential priority |
| --- | --- | --- | --- | --- | --- |
| Management of cardiovascular disease |  |  |  |  |  |
| Management of digestive disease |  |  |  |  |  |
| Management of endocrine disease |  |  |  |  |  |
| Management of respiratory disease |  |  |  |  |  |
| Management of eye, ear, nose, and throat disorders |  |  |  |  |  |
| Management of skin disease |  |  |  |  |  |
| Mental health |  |  |  |  |  |
| Management of common symptoms |  |  |  |  |  |
| Maternal health |  |  |  |  |  |
| Child care |  |  |  |  |  |
| Geriatric care |  |  |  |  |  |

**Part 2. Clinical skills**

| Skill | Not a priority | Low priority | Medium priority | High priority | Essential priority |
| --- | --- | --- | --- | --- | --- |
| Consultation skills and health records writing (SOAP) |  |  |  |  |  |
| Physical examination |  |  |  |  |  |
| Basic first aid |  |  |  |  |  |
| Asepsis |  |  |  |  |  |
| Minor surgery |  |  |  |  |  |
| Interpreting laboratory results |  |  |  |  |  |
| Interpreting radiology reports (computerised tomography scan, ultrasound scan) |  |  |  |  |  |
| Uses and interpretations of ABPM、ECG and holter monitor |  |  |  |  |  |
| Pulmonary function testing |  |  |  |  |  |
| Common examination of the ear, nose and throat |  |  |  |  |  |
| Blood glucose monitoring and insulin injection |  |  |  |  |  |
| Inhalation therapy |  |  |  |  |  |

SOAP: A subjective – Objective – Assessment - Plan medical record notes.

ABPM: Ambulatory blood pressure monitoring.

ECG: Electrocardiogram.

1. **Training format**

What is your preferred training format for continuing medical education?

1. bedside teaching
2. intensive courses such as classroom-based
3. oral presentations such as conferences and lectures
4. online instruction
